# Instrumental variable and colocalization analyses identify endotrophin and HTRA1 as potential therapeutic targets for coronary artery disease

**DOI:** 10.1016/j.isci.2024.110104

**Published:** 2024-05-24

**Authors:** Paul C. Lee, In-Hyuk Jung, Shreeya Thussu, Ved Patel, Ryan Wagoner, Kendall H. Burks, Junedh Amrute, Jared S. Elenbaas, Chul Joo Kang, Erica P. Young, Philipp E. Scherer, Nathan O. Stitziel

**Affiliations:** 1 Center for Cardiovascular Research, Division of Cardiology, Department of Medicine, Washington University School of Medicine, Saint Louis, MO 63110, USA; 2 McDonnell Genome Institute, Washington University School of Medicine, Saint Louis, MO 63108, USA; 3 Touchstone Diabetes Center, University of Texas Southwestern Medical Center, Dallas, TX, USA; 4 Department of Genetics, Washington University School of Medicine, Saint Louis, MO 63110, USA

**Keywords:** Cardiovascular medicine, Techniques in genetics, Quantitative genetics, Biocomputational method, Association analysis

## Abstract

Coronary artery disease (CAD) remains a leading cause of disease burden globally, and there is a persistent need for new therapeutic targets. Instrumental variable (IV) and genetic colocalization analyses can help identify novel therapeutic targets for human disease by nominating causal genes in genome-wide association study (GWAS) loci. We conducted cis-IV analyses for 20,125 genes and 1,746 plasma proteins with CAD using molecular trait quantitative trait loci variant (QTLs) data from three different studies. 19 proteins and 119 genes were significantly associated with CAD risk by IV analyses and demonstrated evidence of genetic colocalization. Notably, our analyses validated well-established targets such as PCSK9 and ANGPTL4 while also identifying HTRA1 and endotrophin (a cleavage product of COL6A3) as proteins whose levels are causally associated with CAD risk. Further experimental studies are needed to confirm the causal role of the genes and proteins identified through our multiomic cis-IV analyses on human disease.

## Highlights

• Cis*-*instrumental variable analyses nominate genes and proteins causal for CAD
• Genetic colocalization nominates shared causal SNPs between expression and CAD
• Phenome-wide MR enables prediction of side effects of modulating targets
• HTRA1 and collagen-VI derived endotrophin may represent therapeutic targets for CAD

## Graphical abstract

**Figure:**
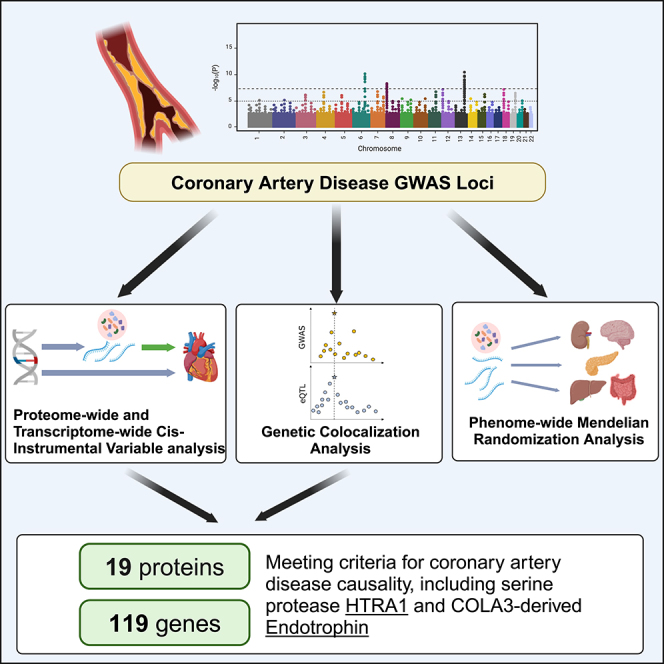

## Introduction

Coronary artery disease (CAD) is a chronic, multifactorial disease and the leading cause of death and disease burden globally.^1^ While effective pharmacological approaches exist to combat CAD, residual risk remains significant due to its complex pathophysiology. As a result, there is a need for novel molecular therapeutic targets that address CAD pathogenesis.^2^ While genome-wide association studies (GWAS) for CAD have identified over 200 loci that may genetically influence disease risk,^3^ the biological mechanisms by which most of these loci act upon disease are unknown, hampering translation toward new therapeutics. This bottleneck has been largely driven by the difficulty in fine-mapping functional variants, nominating causal gene targets within the implicated loci, and validating the functional roles of identified genes.

Integrating GWAS and molecular quantitative trait loci (QTLs) mapping studies may help prioritize causal genes, and several causal inference and instrumental variable analysis methods have been developed to identify gene-trait or protein-trait associations.^4^^,^^5^ For example, drug-target Mendelian randomization (MR) has been widely applied to successfully nominate causal therapeutic targets for conditions ranging from heart failure to COVID-19.^6^^,^^7^^,^^8^ In MR, genetic variants randomly allocated at birth are used as instrumental variables to estimate the causal effect between a genetically determined level of exposure (e.g., plasma protein levels) and an outcome (e.g., disease) that is resistant to confounding, with fulfillment of certain assumptions.^9^ A typical instrument selection procedure involves the selection of genome-wide significant cis-SNPs that are pruned to remove variants in linkage disequilibrium. Similarly, transcriptome-wide association studies (TWAS) and proteome-wide association studies (PWAS) seek to find associations between the genetically predicted transcriptome or proteome, respectively, and the disease trait.^10^ Unlike MR, however, a penalized regression model is typically utilized for polygenic modeling of cis-SNPs that are predictive of molecular traits with relaxed assumptions about causality of individual instruments.^10^ Several recent studies have reformulated TWAS/PWAS (hereafter referred to as “xWAS”) in a probabilistic MR framework where the xWAS can be viewed as a two-stage MR study with critical differences in modeling assumptions and subsequent interpretation.^11^^,^^12^

**Figure 1:**
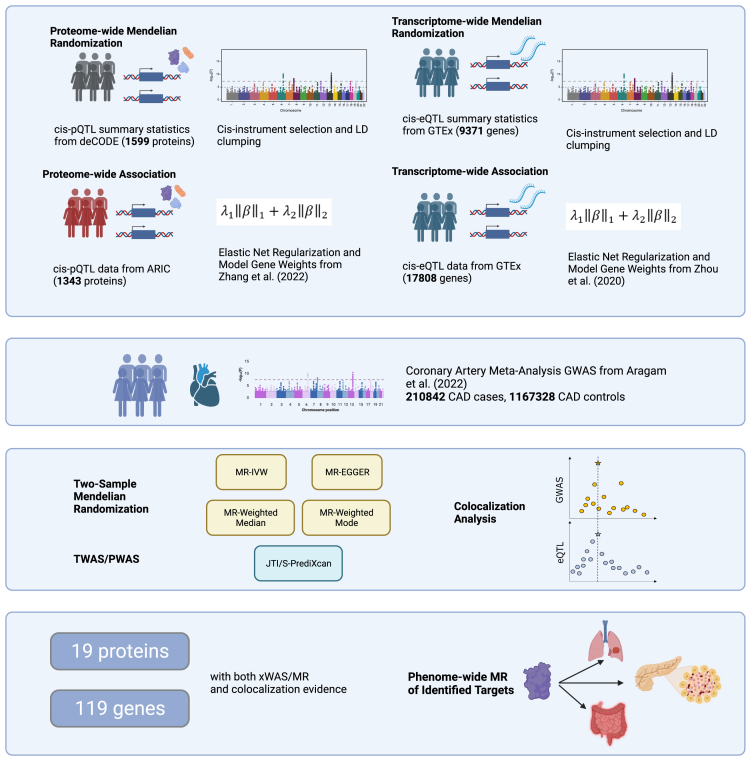
Study overview A graphical overview of the four causal inference methods, data sources, and downstream analyses is shown. Figure created with Biorender.

Previous efforts in causal gene prioritization have mostly focused on applying gene expression-based quantitative trait locus (eQTL) instruments. Plasma protein-based QTL (pQTL) instrumental variable analyses may allow identification of additional causal proteins not identified by eQTL-based methods, given that proteins levels represent a molecular phenotype more “proximal” to the disease trait. Additionally, integrating several different tissue-specific or molecular-trait-specific IVs, as well as causal inference methods, may reveal additional mechanisms as to how these targets may exert their effects on disease. Here, we applied both xWAS and MR methods to multi-tissue genomics and proteomics data to nominate therapeutic targets that may be causal for CAD, leveraging population-wide eQTL and pQTL association data (Figure 1). In an analysis of 20,125 genes and 1,746 plasma proteins measured across three different population-wide studies, we demonstrated that these causal inference methods identify a wide range of known and novel targets which may reflect differences in modeling methods, as well as disparate regulatory mechanisms. We also performed Bayesian colocalization analyses and sensitivity analyses to test the assumptions of the MR models and nominated sets of fine-mapped genetic variants that may mediate the causal relationships. Finally, we evaluated the druggability and potential on-target side effects of therapeutic modulation of our target genes and proteins through a phenome-wide MR of clinical outcomes in the UK BioBank study.

## Results

### Identification of genes and proteins causally associated with CAD

**Figure 2:**
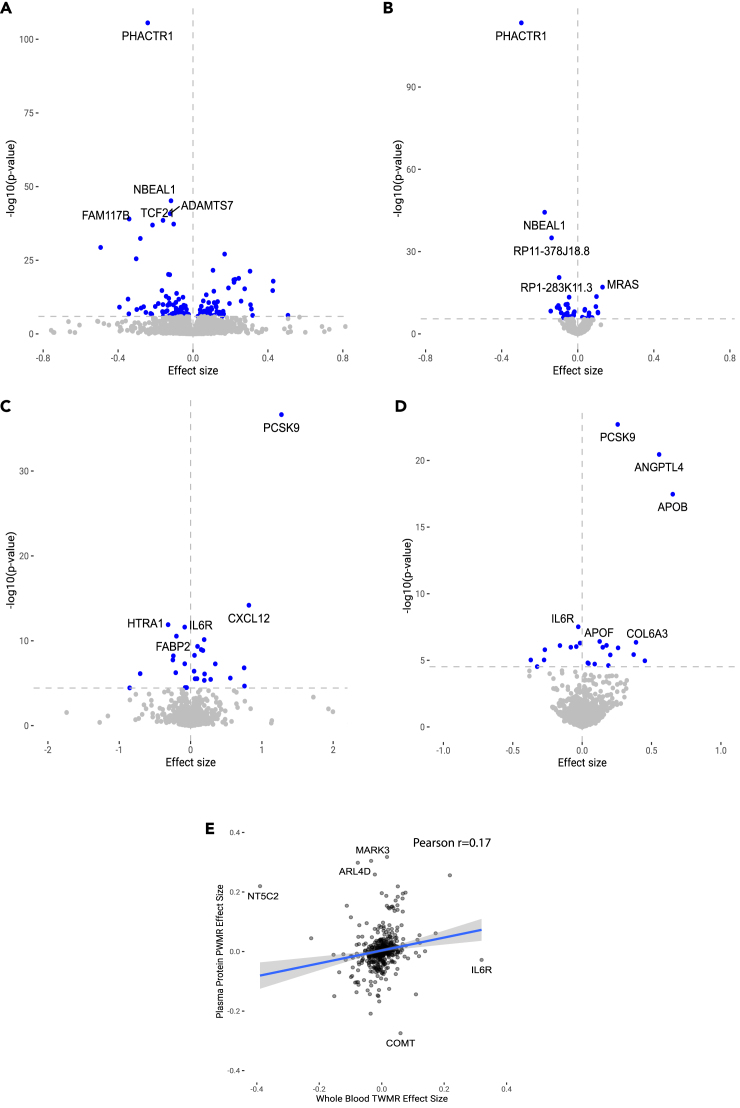
Cis-IV analyses of gene expression and plasma protein levels Individual effect sizes associated statistical significance are shown for results from (A) TWAS based on imputed gene expression in coronary artery, (B) TWMR based on gene expression in coronary artery, (C) PWAS, and (D) PWMR. Blue points indicate significant associations after correcting for multiple testing within each analysis and gene labels indicate the most significant associations in each panel. (E) Correlated effect sizes from results of whole blood TWMR and whole blood PWMR are plotted for each gene. Results with the largest discordance in effect sizes are labeled.

To identify significant associations between gene expression and CAD, we performed a transcriptome-wide association study (TWAS) using the S-PrediXCan method to integrate the meta-analysis CAD GWAS results with the tissue-specific gene expression data from GTEx,^13^ using four tissues (liver, coronary artery, aorta, and whole blood) that were chosen due to their relevance to CAD as well as serving as the possible tissue origin of plasma proteins. For tissue-specific gene expression imputation, we utilized the joint-tissue imputation (JTI) model which leverages cross-tissue similarities in cis-regulatory elements to improve prediction. The TWAS identified 540 significant gene-disease associations across four tissues at a Bonferroni-corrected *p* value threshold of 1.18x10^−6^ (0.05/42,205 gene-tissue pairs), totaling 308 unique candidate genes across all four tissues (Table S2). The most significant gene-disease association was a negative disease association with *PHACTR1* expression in the coronary artery and the aorta (Figure 2A), followed by negative disease associations with *PSRC1, CELSR2, SORT1* expressions in the liver (Table S2). *PSRC1, CELSR2,* and *SORT1* all lie within the same cis-window; the nomination of all three genes is likely due to co-regulation of these genes by overlapping SNP instruments.

As an additional method to nominate causal genes at a transcriptome level, we performed transcriptome-wide MR (TWMR). Unlike a TWAS, which considers non-genome-wide significant SNPs in the cis-locus to generate polygenic transcriptome predictions without consideration of pleiotropy, only genome-wide significant cis-instruments (eQTL *p* values ≤ 5x10^−8^) were used for the TWMR analysis as described in the STAR Methods. Based on these criteria, we generated MR instruments for 15,954 transcripts (9,432 unique genes) spanning the four different tissue types. The TWMR found 248 putative causal gene-disease associations (174 unique genes) across four tissues at a *p* value threshold of 3.13x10^−6^ (0.05/15,954 gene-tissue pairs) (Table S3). Of the 174 unique genes, 113 were also identified by TWAS. Similar to the TWAS, the top nominated gene targets included *PHACTR1* expression in the coronary artery (Figure 2B) and *PSRC1*, *CELSR2*, and *SORT1* expression in the liver (Table S3), with a shared genetic instrument again representing the latter three genes.

Beyond transcript-based analyses which can be obfuscated by shared co-regulation (as highlighted by the overlapping identification of *PCSR1*, *CELSR2*, and *SORT1)*, the incorporation of additional molecular phenotypes such as protein levels has the potential to discover additional causal targets. To identify potential causal protein associations, we used pre-trained models of cis-pQTL SNPs (similar to the TWAS approach) from ARIC^14^ with S-PrediXCan to perform a proteome-wide association study (PWAS) of CAD with plasma proteins. Of 1,343 proteins tested, the PWAS revealed 32 significant plasma protein-disease associations (representing 29 unique proteins) when correcting for multiple testing (Table S4). The top plasma protein targets associated with modified disease risk included PCSK9 and CXCL12 (Figure 2C), both of which have been experimentally and genetically validated as the causal gene in their respective loci.^15^^,^^16^

Finally, we conducted a proteome-wide MR (PWMR) to nominate additional causal plasma proteins, using summary statistics from the recent deCODE pQTL study (*n* = 35,559) to generate cis-pQTL instruments for 1,658 aptamers representing 1,599 unique proteins. We discovered 24 significant plasma protein-disease associations at a *p* value threshold of 3.0 × 10^−5^ (0.05/1658 aptamers), 8 of which were also significant in PWAS (Tables S5 and S6). Plasma protein targets with significant associations with altered disease risk included PCSK9, ANGPTL4, and APOB (Figure 2D), all three of which play well-characterized roles in CAD risk by regulating lipid and triglyceride metabolism.

Although MR selects a small set of genome-wide significant SNPs as instruments, the xWAS family of inference methods allows many SNPs that are in LD with each other and enables polygenic modeling of molecular traits.^11^ Additionally, the surprisingly low shared genetic regulation of plasma proteins and RNA levels raises the question of whether modeling these different molecular traits as exposures will result in different effect estimates.^17^^,^^18^^,^^19^ To assess whether transcriptome- and proteome-wide causal inference methods differed in their predictions, we next explored the concordance in direction and size of effect between the transcriptome- and proteome-based methods. The estimates of disease association were generally similar between xWAS and their corresponding MR methods (e.g., Pearson correlation r = 0.80 for effect sizes from coronary artery TWAS and TWMR, data not shown), indicating that the sparser instrument selection framework utilized in two-sample MR can be used to obtain a similar disease association estimate as the xWAS methods. However, in all four tissues studied there were larger discordances in the disease association estimates between methods utilizing instruments for tissue-specific gene expression versus plasma protein levels with Pearson correlations ranging between r = 0.08 to r = 0.33 (Figures 2E and S1A–S1C). One of the proteins with the biggest discordance in direction included interleukin 6 receptor (IL6R), a cytokine receptor whose soluble form has been previously reported to have a more proinflammatory role in plasma compared to its membrane-expressed counterpart.^20^^,^^21^ Overall, the discordance between tissue expression and plasma protein effects may be driven by both co-regulation and tissue-specific regulation of gene expression by eQTL variants as well as post-translational modification that may be accounted for by pQTL variants as noted in previous studies.^17^

### Colocalization analysis prioritizes IV associations by demonstrating shared genetic association between gene/protein levels and CAD

Valid instrumental variables in MR rely on several key assumptions^22^; horizontal pleiotropy, reverse causality, and linkage disequilibrium can violate these assumptions, resulting in biased MR estimates. After applying Cochran’s Q test to our MR results, we found evidence of heterogeneity in six of the 24 significant PWMR associations and in three of the 242 significant TWMR associations, justifying our choice of a multiplicative random effects model for our primary analysis (Table S7). Only one of the TWMR associations (and none from PWMR) demonstrated a significant intercept term in the MR-EGGER model, indicating that our MR instruments were likely not biased by directional pleiotropy (Table S7).

Partial linkage disequilibrium between different causal variants associated with the exposure and outcome can also bias IV analyses.^21^^,^^23^ In addition, establishing the presence of a shared genetic signal in the exposure and outcome of GWAS can help identify potentially causal SNPs that may be responsible for both traits. Using two probabilistic colocalization methods (coloc^24^ and eCAVIAR^25^), we found that 191 gene-trait or protein-trait associations also demonstrated evidence of colocalization in the cis locus (Table S8), representing a total of 119 unique genes or 19 unique encoded protein products with 2 hits nominated at both gene and protein levels. Approximately one-third (210/616) of our results demonstrated evidence of non-colocalization, suggesting that those MR/xWAS findings may have been driven by LD between different causal SNPs (Table S8). Finally, another third (215/616) of our results did not find evidence of a causal variant for either the exposure or the outcome, potentially due to a lack of statistical power for these colocalization analyses. Overall, there was a high degree of overlap (73/136 unique genes/proteins) between our list of nominated genes and proteins and those identified by previous studies using other causal gene prioritization methods (Table S9).^3^^,^^26^^,^^27^

### Collagen-6-derived peptide endotrophin and serine protease HTRA1 may represent therapeutic targets for CAD

To nominate high priority therapeutic targets for further investigation, we selected proteins that were significantly associated with CAD in PWAS and PWMR with evidence of shared causal genetic association (Table S9). We chose to focus on plasma protein targets as they represent a molecular phenotype downstream of transcription that may be more readily targeted by therapeutic strategies such as neutralizing antibodies.^28^^,^^29^ Previous large-scale drug target MR studies have shown that a similar selection strategy can result in an enrichment of successful clinical trial targets.^21^^,^^30^ Five proteins satisfied the aforementioned criteria: PCSK9, IL6R, COL6A3, HTRA1, and PPCS. PCSK9 is an established therapeutic target for CAD,^31^ and clinical trials of IL-6 inhibition in CAD are ongoing.^32^ PPCS is an enzyme involved in CoA synthesis whose inhibition may plausibly affect downstream mevalonate levels and cholesterol biosynthesis.^33^^,^^34^^,^^35^ Therefore, we decided to focus on COL6A3 and HTRA1 as potential regulators of coronary artery disease in humans.

**Figure 3:**
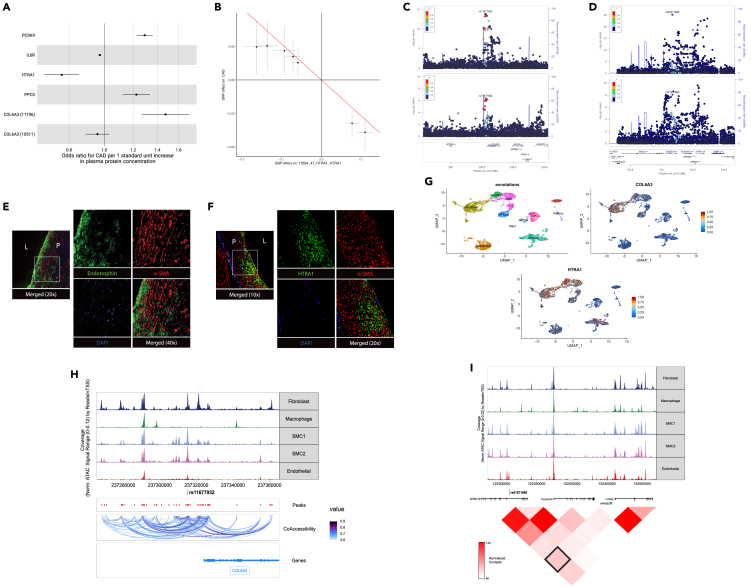
Endotrophin and HTRA1 causally associate with CAD (A) Odds ratio of CAD for genetically encoded one standard deviation increase in plasma protein levels are shown for the most significant PWMR results. COL6A3 is independently measured by two aptamers (11196 and 10511). (B) Estimated effect (with 95% confidence intervals) of each variant included in the Mendelian randomization for HTRA1 levels and CAD risk. Regional association plots showing colocalization evidence between protein level (top) and CAD associations (bottom) for (C) COL6A3 (colocalization posterior probability = 0.98) and (D) HTRA1 (colocalization posterior probability = 0.86). Immunohistochemistry in human aortic tissue demonstrating expression of (E) endotrophin and (F) HTRA1 in green with co-staining for ɑ-SMA and DAPI to label smooth muscle cells and nuclei, respectively. The outlined area in the left of each panel indicates the region magnified in the panels to the right. L = aortic lumen. P = atherosclerotic plaque. (G) Single cell expression of COL6A3 and HTRA1 in human coronary artery. (H) Cell-type specific chromatin accessibility surrounding the lead CAD disease risk variant in the COL6A3 locus. (I) Cell-type specific chromatin accessibility (top) and Hi-C contact map at 40kB resolution (bottom) surrounding the lead CAD disease risk variant in the HTRA1 locus. The black box highlights contact between region surrounding rs61871680 and the HTRA1 promoter region.

COL6A3 is a subunit of collagen VI, a component of the extracellular matrix produced by fibroblasts and adipocytes.^36^^,^^37^ In PWMR, we found that genetically predicted plasma levels of circulating COL6A3 (as measured by aptamer 11196) were causally associated with increased risk of CAD (Figure 3A); COL6A3 was also significantly positively associated with CAD in the PWAS (*p* = 5.3x10^−8^). The likely causal variant identified in colocalization was rs11677932, which was both the lead pQTL for the protein level association and the disease association in the locus (Figure 3C). Notably, the risk increasing allele of rs11677932 is also associated with increased *COL6A3* total transcript levels in both GTEx and Stockholm-Tartu Atherosclerosis Reverse Networks Engineering Task (STARNET) studies in the aorta (GTEx Aorta *p* = 1.6x10^−6^ and STARNET Aorta *p* = 6.91x10^−6^).^38^^,^^39^ Although *COL6A3* is highly expressed in the adipose tissue and has been shown to be associated with metabolic dysregulation, the risk allele was not associated with altered levels of *COL6A3* transcripts in subcutaneous (*p* = 0.4) or visceral adipose (*p* = 0.6) in GTEx, indicating the genetic regulation may be specific to vascular tissue.

Surprisingly, in the PWMR we found that a different aptamer targeting plasma COL6A3 (aptamer 10511) had no association with CAD (*p* = 0.25; Figure 3A). Further investigation into the specific epitopes targeted by the two different aptamers revealed that aptamer 10511 targets the N-terminal region of COL6A3 (amino acids 26–1036) while aptamer 11196 targets the C-terminal Kunitz domain of COL6A3 (amino acids 3108–3165), which is known to be readily cleaved to form the matrikine endotrophin.^40^ These discordant MR results suggest that the circulating or tissue-specific levels of endotrophin, but not the total COL6A3 protein, modulates coronary artery disease risk.

While the PWMR analysis is based on genetically predicted circulating levels of endotrophin, we further explored whether endotrophin could play a more local role in the coronary artery to promote atherosclerosis. Immunohistochemistry demonstrated that endotrophin is highly expressed near the fibrous cap of atherosclerotic plaques in both humans and mice (Figures 3E and S2A). Analyses of single-cell expression and chromatin accessibility suggest that endotrophin is highly expressed in phenotypically modulated smooth muscle cells and fibroblasts within the coronary artery and that the causal SNP lies at an accessible region within those two cell types (Figures 3G and 3H).^41^^,^^42^ Together, these results suggest a plausible hypothesis that the local production and release of endotrophin in the coronary artery may promote atherogenesis.

Another nominated target was HTRA1, a secreted serine protease with a wide range of identified substrates such as TGF-beta, bone morphogenic protein 4 (BMP4), and growth differentiation factor 5 (GDF5).^43^ Increasing levels of circulating HTRA1 were causally associated with decreased risk of coronary artery disease in our PWMR, suggesting that HTRA1 may play a protective role in disease (Figures 3A and 3B). Colocalization evidence suggests that the pQTL and CAD results are likely driven by rs61871680, which lies upstream of *HTRA1* in an intronic region of *BTBD16* (Figure 3D). While rs61871680 is also an eQTL variant for *BTBD16* expression in GTEx in both the aorta and the coronary artery, the association for *BTBD16* is not supported by colocalization evidence (H3 > 0.95).^38^ Surprisingly, rs61871680 is not an eQTL variant for HTRA1 expression in any tissue in GTEx.

Immunohistochemistry in atherosclerotic plaques from humans and mice revealed that HTRA1 expression was mostly localized to the neointima (Figures 3F and S2B). Single-cell expression data suggested that HTRA1 is widely expressed in different vascular cell types including fibroblasts, SMCs, ECs, and myeloid cells (Figure 3G).^41^ Interestingly, the colocalized rs61871680 variant lies in an endothelial cell-specific marker peak identified in the single-nucleus ATAC-seq data (GRCh38 chromosome 2: 122310837–122311237) that is significantly more accessible in endothelial cells compared to other cell types (*p* = 6.1x10^−8^ for endothelial cells compared to all other cell types), suggesting that the disease phenotype may be specific to endothelial cells (Figure 3I).^42^ Additionally, genome-wide Hi-C data in human embryonic vein endothelial cells identified a 25 kB region containing this SNP as a putative enhancer domain based on enrichment of chromatin contact with the promoter region of *HTRA1* (Figure 3I).^44^^,^^45^ Given the well-characterized roles of TGF-beta family proteins in modulating smooth muscle cell phenotypes and promoting endothelial-mesenchymal transition (EndoMT) in atherosclerosis, it is possible that the production of HTRA1 by smooth muscle and endothelial cells plays a protective role by limiting excessive TGF-beta signaling.^46^^,^^47^^,^^48^

### Phenome-wide MR and drug repositioning analyses predict on-target side effects and inform therapeutic strategies

**Figure 4:**
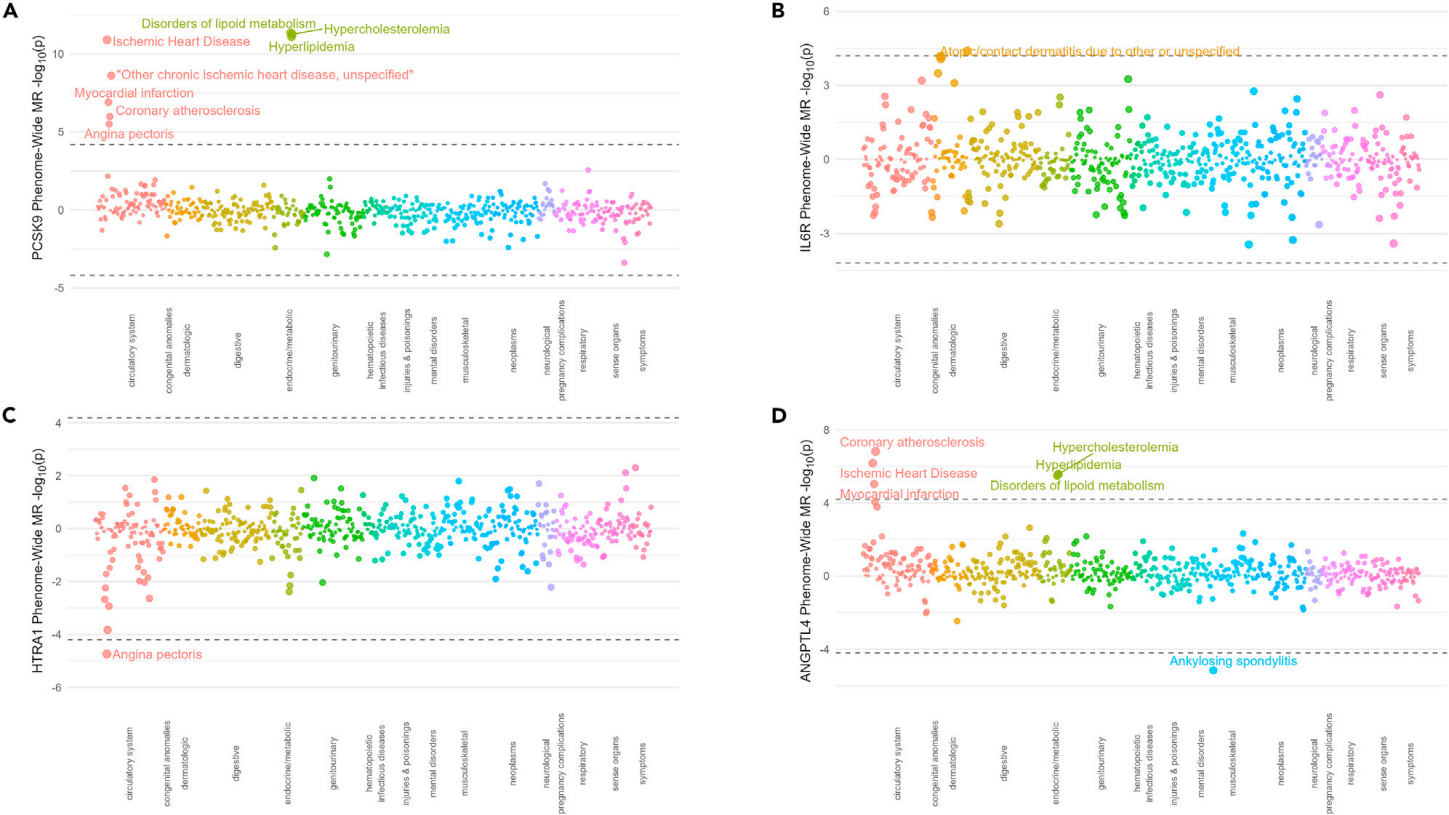
Phenome-wide mendelian randomization Phenome-wide MR across clinical PheCodes in the UK Biobank with plasma protein levels of (A) PCSK9, (B) IL6R, (C) HTRA1, or (D) ANGPTL4.

We next performed a phenome-wide MR with 785 clinical traits from UK Biobank to help predict whether the targeting proteins of interest may have unintended on-target effects. We used 118 significant gene-CAD or protein-CAD associated MR instruments, excluding the results from xWAS that may not have suitable instruments for downstream MR analyses. As expected, we found that many (49/118) of the targets were causally associated with on-target traits related to cardiovascular disease (Table S10). Similarly, 13 targets were also associated with metabolic phenotypes such as lipid-related traits. In keeping with previously reported safety profiles, genetically predicted reduction of plasma PCSK9 levels was causally associated with plasma lipid levels and atherosclerotic risk without significant associations for other phenotypes (Figure 4A).^31^^,^^34^ Surprisingly, neither COL6A3, PPCS, nor IL6R levels were causally associated with increased risk of coronary atherosclerosis in this cohort, although higher levels of circulating IL6 receptor were causally associated with increased risk of atopic dermatitis (Figure 4B). The genetically predicted increase in plasma HTRA1 levels was associated with reduced risk of angina pectoris (Figure 4C), consistent with the PWMR results. Among other therapeutic targets currently in investigation for CAD, we found that lower levels of ANGPTL4 were causally associated with increased risk of ankylosing spondylitis (Figure 4D) in addition to expected associations with cardiovascular and lipid phenotypes, which is consistent with a previous finding linking the ANGPTL4 E40K partial loss-of-function variant with increased risk of ankylosing spondylitis.^49^

Previous work suggested that target-disease associations supported by MR are more likely to succeed in FDA approval.^21^^,^^50^ Motivated by this, we asked if there were currently approved compounds targeting the genes and proteins identified in this study that may be suitable for drug repurposing. 32 and 44 genes and proteins with both xWAS/MR and colocalization evidence were found in either the DrugBank^51^ (Table S11) or the druggable genome database (Table S12),^52^ respectively, representing therapeutic targets that are either drugged or considered druggable by approved pharmaceuticals or by agents in various stages of clinical development. 15 of these were in the druggable genome tier 1, indicating a protein with efficacious drug candidates that have already been approved for clinical use as well as candidates that are currently in clinical trials.

## Discussion

Instrumental variable analyses using cis-genetic instruments represent a promising approach to identify causal genes and protein targets that may be suitable for therapeutic intervention. Here, we performed causal inference analyses for CAD with 20,125 genes and 1,746 circulating proteins, identifying 119 genes and 19 proteins that may play a causal role in CAD. Our results include well-characterized regulators of plasma lipoprotein metabolism that are existing drug targets for coronary artery disease (e.g., PCSK9), as well as experimentally uncharacterized targets that have not been directly linked to coronary artery disease (e.g., endotrophin, PPCS, and HTRA1).^53^^,^^54^ Many of our identified genes and proteins are classified in tiers I–III of the druggable genome resource, indicating that they are promising targets for therapeutic intervention.

To date, our study represents one of the largest systematic investigations of causal roles of genes and proteins in coronary artery disease utilizing cis*-*IV analyses and colocalization. Our study leveraged summary statistics from larger and more comprehensive proteomic and transcriptomics datasets than those previously utilized in MR studies, allowing us to refine and add to previous results with greater statistical power.^7^^,^^21^^,^^55^^,^^56^^,^^57^ For example, the data from the deCODE group allowed us to construct suitable MR instruments for ∼1,600 unique proteins, exceeding the largest prior study of 1,002 proteins.^21^ Additionally, while previous studies have used tissue-specific GTEx and STARNET eQTLs, our analyses utilized larger CAD GWAS outcomes, different IV methods, and integrated plasma pQTLs. This allowed us to identify additional targets and draw broader insights regarding tissue specificity and post-transcriptional regulation of the nominated targets.^26^^,^^27^

Many of the causal genes and proteins we identified are existing or nominated therapeutic targets or are involved in CAD pathobiological pathways. These include targets such as ANGPTL4, APOB, IL6R, and PCSK9, all of which have well-known roles in lipoprotein metabolism, inflammation, and CAD risk.^32^^,^^34^^,^^58^ Our phenome-wide MR analyses suggest that PCSK9 inhibition should be well-tolerated without substantial side effects, consistent with clinical trials of PCSK9 inhibitors and previous MR studies of PCSK9 levels.^59^^,^^60^^,^^61^ Additionally, we nominate COL6A3-derived peptide endotrophin and serine-protease HTRA1 as potential regulators of CAD risk. COL6A3 is a subunit of collagen VI trimer that has been previously implicated in a variety of phenotypes including cell senescence, ascending aorta size, and obesity.^62^^,^^63^^,^^64^ Similarly, endotrophin is a COL6A3-derived matrikine whose plasma levels have been associated with a variety of diseases including adverse cardiovascular events, heart failure, type 2 diabetes, and cancer.^65^^,^^66^^,^^67^^,^^68^^,^^69^ The pro-inflammatory and pro-fibrotic roles of endotrophin in tumor and adipose tissues have been extensively studied, but the mechanism by which endotrophin may modulate atherosclerotic risk is unclear.^65^^,^^70^ Although it is highly expressed in other tissues such as adipose, endotrophin may also promote local inflammation in the arterial wall as it is expressed in the neointima and the CAD risk variant is a vascular tissue-specific eQTL.^38^^,^^66^^,^^71^ Further studies are needed to examine whether endotrophin neutralization may reduce the risk of CAD.

Similarly, HTRA1 is a secreted serine protease with antagonistic action on a variety of signaling molecules, including the TGF-beta family of proteins.^43^ Loss-of-function mutations in *HTRA1* cause cerebral autosomal recessive arteriopathy with subcortical infarcts and leukoencephalopathy (CARASIL),^72^ and *HTRA1* is a GWAS locus for age-related macular degeneration.^73^^,^^74^ Additionally, plasma levels of HTRA1 have been previously shown to associate with lower risk of type 2 diabetes.^75^ Here, using pQTL data we nominate increased HTRA1 as a possible protective regulator of CAD. The role of TGF-beta signaling in atherosclerosis is complex, and it is possible that increased expression of HTRA1 by phenotypically modulated smooth muscle cells and endothelial cells inhibits atherogenic TGF-beta signaling, thereby preventing neointima formation and pathogenic EndoMT similar to neointima formation seen in CARASIL.^48^ Further *in vivo* studies are needed to clarify the mechanism by which HTRA1 may influence atherosclerosis.

The uncertainty of whether normalized RNA and soluble protein levels accurately reflect expression and activity in disease relevant tissue and cell contexts poses a challenge to interpreting the results of locus-based proteome-wide and transcriptome-wide IV analyses.^56^ For example, it is possible for the soluble and membrane-bound forms of a protein to have disparate biological roles which may obfuscate the direction of causal effects when plasma protein levels are used as the exposure. This is highlighted in our study by the discordant results for proteins such as IL6R. Additionally, these findings are consistent with a previous multivariable-MR mediation analysis which concluded that protein expression mediates a small proportion of the transcript-to-disease causal estimate.^30^ It is also possible that the plasma protein levels are not an accurate reflection of abundance or activity of the protein within the cell for proteins that are not secreted into the plasma. However, the large size of modern proteomics studies, the ability of pQTL instruments to pinpoint causal proteins not implicated by eQTL instruments, and ability to capture post-translational modifications such as peptide cleavage make proteome-wide IV analyses an attractive option for gene prioritization.

In conclusion, our study utilizes a set of IV analyses methods to identify putative causal proteins and genes in CAD GWAS loci that may serve as promising candidates in both functional follow-up experiments as well as pharmaceutical investigation. As multiomic QTL discovery in tissue, disease, and cell-specific contexts becomes more and more feasible, such IV analyses methods are expected to continue enhancing our mechanistic understanding of GWAS loci.

### Limitations of the study

Our study has several important limitations. First, the “missing regulation” problem and the high error rate of identifying causal genes in GWAS loci using molecular QTL variants have been well-described, and it is likely that our analysis suffers from similar limitations due to hypothesized inherent differences in QTL and GWAS variants.^50^^,^^76^^,^^77^^,^^78^ Second, it is possible that protein-altering variants (PAVs) included as instruments in our studies alter aptamer binding, resulting in inaccurate measurement of protein concentrations. Although some previous proteome-wide MR studies excluded PAVs (and SNPs in high LD with PAVs), we chose to include PAVs as they may still result in a meaningful biological alteration of plasma protein concentration.^79^ Third, our QTL association and outcome analyses were based on studies consisting primarily of participants with European ancestry. It is possible that ancestry-specific effects on both protein levels and disease are not captured here. Fourth, it is impossible to completely exclude the possibility that horizontal pleiotropy and linkage disequilibrium between different causal SNPs biased the MR. This problem is particularly exacerbated by single-SNP MR or MR with a few SNPs, where a single pleiotropic SNP could strongly influence the causal effect estimate. Finally, despite statistical methods to control for potential pleiotropy, cis-IV analyses are unable to distinguish between genes and proteins that are co-regulated by the same cis variants (e.g., the nomination of PSRC1, CELSR2, and SORT1). Further integration of multiomic datasets (eQTL/transcriptomics, mQTL/epigenomics, etc.) in various tissues and cell types for causal gene prioritization may help pinpoint causal genes as well as help understand how the target gene or protein products are expressed and regulated.^30^^,^^80^

## STAR★Methods

### Key resources table

**Table:** 

REAGENT or RESOURCE	SOURCE	IDENTIFIER
**Antibodies**		

Anti-HTRA1 antibody	ProteinTech	Cat# 55011-1-AP; RRID:AB_10859830
Anti-αSMA-Cy3 antibody	Sigma Aldrich	Cat# C6198; RRID:AB_476856
Donkey anti-rabbit IgG secondary antibody, Alexa Fluor 488	Invitrogen	Cat# A-21206; RRID:AB_2535792

**Deposited data**		

deCODE pQTL summary statistics	Ferkingstad et al.^81^	https://www.decode.com/summarydata/
ARIC pQTL summary statistics	Zhang et al.^14^	http://nilanjanchatterjeelab.org/pwas/
ARIC PWAS models	Zhang et al.^14^	http://nilanjanchatterjeelab.org/pwas/
GTEx eQTL summary statistics	GTEx Consortium^38^	https://gtexportal.org/home/downloads/adult-gtex/overview
GTEx JTI TWAS models	Zhou et al.^82^; Zenodo	https://zenodo.org/records/3842289
CAD GWAS summary statistics	Aragam et al.^3^; GWAS catalog	GCST90132315
UKBB binary phenome summary statistics	Zhao et al.^83^	https://pheweb.sph.umich.edu/
DrugBank	Wishart et al.^51^	https://go.drugbank.com/
Druggable Genome	Finan et al.^52^	N/A
Human coronary artery single cell RNA-seq data	Wirka et al.^41^	GEO: GSE131778
Human coronary artery single nucleus ATAC-seq data	Turner et al.^42^	GEO: GSE175621
ENCODE PSYCHIC data	Ron et al.^44^	https://www.cs.huji.ac.il/w∼tommy/PSYCHIC/

**Software and algorithms**		

R v.4.1.3	R	N/A
TwoSampleMR v.0.5.6	Hemani et al.^84^	https://mrcieu.github.io/TwoSampleMR/
MendelianRandomization v.0.6.0	Yavorska et al.^85^	https://cran.r-project.org/web/packages/MendelianRandomization/index.html
Ggplot2 v.3.4.1	Wickham et al.^86^	https://cran.r-project.org/web/packages/ggplot2/index.html
Coloc v.5.1.0	Giambartolomei et al.^24^	https://cran.r-project.org/web/packages/coloc/index.html
eCAVIAR v.2.0.0	Hormozdiari et al.^25^	https://github.com/fhormoz/caviar/tree/master
LocusZoom	Pruim et al.^87^	http://locuszoom.org/
Plaqview	Ma et al.^88^	https://plaqview.uvadcos.io/
Seurat v.4.0.0	Hao et al.^89^	https://cran.r-project.org/web/packages/Seurat/index.html
ArchR v.1.0.1	Granja et al.^90^	https://www.archrproject.com/
3D Genome Browser	Wang et al.^91^	http://3dgenome.fsm.northwestern.edu/
Zen	Zeiss	https://www.zeiss.com/microscopy/en/products/software/zeiss-zen.html
Biorender	Biorender	https://www.biorender.com

### Resource availability

#### Lead contact

Further information and requests for resources and reagents should be directed to and will be fulfilled by the lead contact, Dr. Nathan Stitziel (nstitziel@wustl.edu).

#### Materials availability

This study did not generate new unique reagents.

#### Data and code availability

• This paper analyzes existing, publicly available data. All results are published in the main text and Supplementary Tables. Other raw data will be shared by the lead contact upon reasonable request.
• This paper does not report original code.
• Any additional information required to reanalyze the data reported in this work is available from the lead contact upon request.

### Method details

#### Genome-wide association study data

We obtained summary level statistics for European American participants from the genotype-tissue expression project (GTEx) (https://gtexportal.org/home/)^38^ to create eQTL instruments using methods described below. These summary statistics were generated by GTEx using gene expression levels that were inverse normalized across samples as input to FastQTL with covariates of PEER factors, whole-genome sequencing platform and protocol, genotype principal components, and donor sex at an FDR threshold of 0.05.

pQTL instruments were created by methods described below using summary level statistics from the deCODE pQTL study (https://www.decode.com/summarydata/).^81^ The deCODE study included plasma protein levels measured by 4,907 aptamers in 35,559 Icelanders using the SomaScan multiplex aptamer assay. In the deCODE study, protein expression levels were rank inverse-normalized across samples, and age, sex, and sample age were all included as covariates in a linear mixed model.

To conduct the proteome-wide association study, we obtained results of a pre-trained PWAS model previously generated in the Atherosclerosis Risk in Communities (ARIC) cohort.^14^ This model was previously generated using TWAS/FUSION method for plasma proteins measured by 1,350 SomaScan aptamers with non-zero cis-SNP heritability in 7,213 individuals of European ancestry.^92^

For CAD genetic associations, we used summary statistics from a recent CAD GWAS meta-analysis^3^ consisting of 181,522 CAD cases and 984,168 controls. To better match the genetic ancestry of individuals involved in the gene and protein level association studies, we only included the European ancestry sub-study summary statistics. Participant characteristics and case-control definitions of all sub-studies included in the meta-analysis have been previously described.^3^

To perform the phenome-wide Mendelian randomization, we obtained previously published summary statistics for 784 binary phenotypes from the UK Biobank (https://www.leelabsg.org/resources)^83^ based on individuals of European ancestry. Further details on the generation of these results have been previously described.

#### Instrumental variable analyses

The IV analyses involved four different methods: transcriptome-wide and proteome-wide associations with S-PrediXcan along with transcriptome-wide and proteome-wide Mendelian randomization with TwoSampleMR^84^^,^^93^^,^^94^ using exposure and outcomes data described above.

For transcriptome-wide association, we applied Joint Tissue Imputation (JTI) prediction models and S-PrediXcan to identify causal genes in CAD GWAS loci based on tissue-specific eQTLs from GTEx.^82^ Broadly, TWAS models are gene expression imputation models built using Elastic Net regression. JTI models utilize similarities in regulatory elements and gene expression patterns across tissues to further predict tissue-specific expression by “borrowing” information from other tissues; as a result, JTI models are able to nominate more causal genes than PrediXcan models by improving gene level prediction performance. We obtained JTI models that were pre-trained on GTEx v8 data with flexible cis*-*window sizes for liver, coronary artery, aorta, and whole blood (https://zenodo.org/record/3842289). We then used S-PrediXcan with the model weights from the JTI pre-trained model and CAD GWAS summary statistics.^3^

For proteome-wide association, we applied S-PrediXcan on a PWAS prediction model previously generated on plasma protein pQTLs from ARIC.^14^ We obtained pre-trained pQTL data from 7,213 European American individuals from ARIC (https://jh-pwas.s3.amazonaws.com/packages/PWAS_EA.zip) and used a custom script to convert these into a format compatible with S-PrediXcan.^93^^,^^94^

For transcriptome-wide Mendelian randomization, we downloaded the full cis-association summary statistics for GTEx v8 for coronary artery, aorta, liver, and whole blood as well as a list of significant cis*-*eGenes from those tissues from the GTEx portal. To generate cis-instruments, we selected genome-wide significant (5x10^-8^) SNPs within 1MB of transcription start sites for each significant cis-eGene in each tissue. To minimize the effect of pleiotropy, we excluded SNPs that were associated with six or more transcripts in cis at a level of genome-wide significance. SNPs from the major histocompatibility complex (MHC) region (chr6:28510120-33480577) were also excluded due to the high LD complexity in the region. The cis-SNPs were then pruned using PLINK (v1.90) at an r^2^ threshold of 0.1 based on the 1000 Genomes European ancestry population to obtain near-independent instruments. For the primary MR analysis, inverse-variance weighted (IVW) method accounting for residual correlation due to LD was applied to calculate MR association estimates between gene expression and CAD outcomes.^85^ The fixed effect IVW regression method was used when there were two or three SNPs that were available to be used as instruments, and Wald’s ratio method was used to estimate the causal effect when there was only one SNP available to be used as an instrument.^95^ If there were more than three IVs available, the multiplicative random-effect IVW method correcting for residual LD was utilized.

For proteome-wide Mendelian randomization, we obtained cis-pQTL association summary statistics for proteins that had at least one significant cis-pQTL association in the previously published deCODE pQTL study.^81^ Transcription start sites were identified for genes encoding each protein from Ensembl version 106 by matching gene names, resulting in 1,599 unique proteins that were tested in cis-MR analyses. For instrument selection, cis-pQTL SNPs within 1Mb of the transcription start site at a level of genome-wide significance were selected and pruned with an r^2^ threshold of 0.1. SNPs that associated with six or more proteins in cis or were located within the MHC region were excluded. Subsequent MR analyses were performed as described above.

For phenome-wide Mendelian randomization, we utilized the same instrument selection strategy as the transcriptome-wide or proteome-wide Mendelian randomization. Association statistics for binary phenotypes from UKBB were harmonized, and MR methods were applied as described above.

Possible sources of bias on the MR effect estimates include reverse-causality, horizontal pleiotropy, and LD.^22^ To account for reverse-causality in our MR estimates, we performed Steiger filtering test at the instrument level, which removes IVs that explain more variation in the outcome than the exposure. To assess the presence of balanced pleiotropy (heterogeneity within MR instruments), Cochran’s Q statistic was calculated for variant-level causal estimates. To test the robustness of our primary MR method, three additional MR methods (MR-EGGER, Weighted Median, Weighted Mode) were applied and effect size estimates were compared.

For all IV analyses, multiple testing correction was applied using Bonferroni adjusted p-value threshold accounting for either the number of genes or proteins tested. All statistical analyses were performed using R (v. 4.1.3). MR analyses were carried out using R packages *TwoSampleMR* (v.0.5.6) and *MendelianRandomization* (v.0.6.0).^84^^,^^85^ Figures related to IV analyses were generated using *ggplot2* (v. 3.4.1).^86^

#### Colocalization analyses

Using the list of genes and proteins that were significant in the IV analyses, we performed colocalization analysis to identify shared genetic associations between the eQTL/pQTL association studies and the coronary artery disease GWAS. Given the possibility of multiple causal signals in both exposure and outcome, two different types of enumeration colocalization models were employed: coloc method^24^ (implemented using R package *coloc* v. 5.1.0), which assumes that there is at most one causal variant per trait, and eCAVIAR (v.2.0.0),^25^ which relaxes that assumption to allow multiple causal variants per trait. Either locus-level colocalization posterior probability PP.H4 > 70% (coloc.abf) or variant-level colocalization posterior probability CLPP >1% (eCAVIAR) with default priors were considered as evidence for colocalization. Locus-level colocalization PP.H3 > 70% was considered as evidence for distinct causal variants in the locus, and low probability of either H3 or H4 hypotheses was interpreted as a lack of association with either exposure or outcome traits. We allowed for three maximum causal variants in eCAVIAR due to computational constraints; the CLPP cutoff was chosen based on simulation data suggesting that method has an acceptably low false positive rate and outperforms other colocalization methods at this threshold.^25^ Figures related to colocalization were generated using LocusZoom (http://locuszoom.org/).^87^

#### Immunohistochemistry staining and imaging

Immunohistochemistry studies were performed in paraffin embedded sections of human coronary artery or frozen sections of mouse aortic tissues of 5uM thickness. Deparaffinization was performed by baking the slides at 60°C for 1 hour, incubating the sections in xylene twice for 5 minutes, and incubating twice in ethanol for 2 minutes. Slides were air dried at room temperature for 1 hour prior to hydration in phosphate-buffered saline (PBS) for 10 minutes. After heat-induced antigen retrieval in retrieval buffer for 5 minutes (frozen) or 15 minutes (paraffin), the slides were rinsed in water for 15 seconds and incubated in ethanol for 3 minutes. Following another air-dry step at room temperature, sections were permeabilized with PBS containing 0.5% TritonX-100, washed three times with PBS, and subsequently blocked in 5% goat serum with PBS containing 0.5% TritonX-100 for 30 minutes. Slides were then incubated with anti-endotrophin serum (1:200), anti-smooth muscle actin (1:500), anti-HTRA1 (1:200, ProteinTech) antibodies overnight at 4°C. Following three washes in PBS with 0.1% Tween-20 (PBS-T), sections were incubated with secondary antibodies for 30 minutes at room temperature. Following another set of three washes in PBS-T, slides were mounted with ProLong Gold Antifade mounting solution for DAPI counterstaining. Confocal images were obtained on the Zeiss LSM 700 laser scanning microscope and analyzed using the Zeiss Zen (v.3.7) software.

#### Single-cell and Hi-C analyses

To characterize the cell-type specific distribution of candidate genes, we obtained raw data from a previously published single cell RNA-seq experiment (GEO: GSE131778^41^). Quality control was performed to exclude cells with percent mitochondrial genes <5%, nFeature_RNA >500, nFeature_RNA<3500, and nCount_RNA<20000. Data was normalized using SCTransform from Seurat package (v.4.0.0) regressing out mitochondrial counts.^89^ Dimensional reduction using PCA was calculated and UMAP embedding was generated using 50 components. Different clustering resolutions and differential gene expression using the ‘FindAllMarkers’ function was used to annotate cell types using canonical marker genes. FeaturePlot was used to visualize genes of interest in the UMAP embedding. To generate pseudobulk assay for transposase-accessible chromatin sequencing (ATAC-seq) tracts, processed files from Turner et al.^42^ were downloaded from Plaqview (https://www.plaqview.com/data).^88^ ‘PlotBrowserTrack’ function from ArchR (v.1.0.1) was used to generate tracks for author-annotated fibroblasts, macrophages, smooth muscle cells, endothelial cell subtypes.^90^ The ‘getMarkerFeatures’ function was used to identify cell marker peaks accounting for TSS enrichment and unique fragments per cell.

We used the 3D genome browser (http://3dgenome.org)^31^ to generate contact maps of region surrounding the fine-mapped CAD GWAS SNP and the *HTRA1* promoter region from previously published Hi-C data.^45^^,^^91^ Pre-calculated promoter-enhancer prediction data in HUVECs using PSYCHIC were downloaded to identify 25kB region surrounding the fine-mapped SNP as a putative enhancer for *HTRA1*.^44^

### Quantification and statistical analysis

Statistical analyses were conducted using R. Detailed information regarding statistical methods for each analysis can be found in the method details.

## Supplemental information

Document S1. Figures S1 and S2

Table S1. Genome-wide association studies used for Mendelian randomization analyses, related to Figure 1

Table S2. Transcriptome-wide association study across four tissues, related to Figure 2

Table S3. Transcriptome-wide Mendelian randomization analysis across four tissues, related to Figure 2

Table S4. Plasma protein-wide association study, related to Figure 2

Table S5. Plasma proteome-wide Mendelian randomization analysis, related to Figure 2

Table S6. Significant results across four different methods, related to Figure 2

Table S7. Heterogeneity analysis and egger intercept test for MR Hits, related to Figure 2

Table S8. Colocalization analysis for MR/TWAS/PWAS hits, related to Figures 2 and 3

Table S9. Evidence for 136 unique gene/proteins with both Cis-IV and colocalization evidence, related to Figures 2 and 3

Table S10. Phenome-wide MR for significant TWMR/PWMR hits, related to Figure 4

Table S11. Drugbank query results for top MR hits, related to Figure 4

Table S12. Druggable genome classification for top MR hits, related to Figure 4

## References

[1] Roth G.A., Mensah G.A., Johnson C.O., Addolorato G., Ammirati E., Baddour L.M., Barengo N.C., Beaton A.Z., Benjamin E.J., Benziger C.P. (2020). Global Burden of Cardiovascular Diseases and Risk Factors, 1990–2019: Update From the GBD 2019 Study. J. Am. Coll. Cardiol..

[2] Fredman G., Ozcan L., Tabas I. (2014). Common Therapeutic Targets in Cardiometabolic Disease. Sci. Transl. Med..

[3] Aragam K.G., Jiang T., Goel A., Kanoni S., Wolford B.N., Atri D.S., Weeks E.M., Wang M., Hindy G., Zhou W. (2022). Discovery and systematic characterization of risk variants and genes for coronary artery disease in over a million participants. Nat. Genet..

[4] Schadt E.E., Lamb J., Yang X., Zhu J., Edwards S., GuhaThakurta D., Sieberts S.K., Monks S., Reitman M., Zhang C. (2005). An integrative genomics approach to infer causal associations between gene expression and disease. Nat. Genet..

[5] Cano-Gamez E., Trynka G. (2020). From GWAS to Function: Using Functional Genomics to Identify the Mechanisms Underlying Complex Diseases. Front. Genet..

[6] Holmes M.V., Ala-Korpela M., Smith G.D. (2017). Mendelian randomization in cardiometabolic disease: challenges in evaluating causality. Nat. Rev. Cardiol..

[7] Henry A., Gordillo-Marañón M., Finan C., Schmidt A.F., Ferreira J.P., Karra R., Sundström J., Lind L., Ärnlöv J., Zannad F. (2022). Therapeutic Targets for Heart Failure Identified Using Proteomics and Mendelian Randomization. Circulation.

[8] Palmos A.B., Millischer V., Menon D.K., Nicholson T.R., Taams L.S., Michael B., Sunderland G., Griffiths M.J., Hübel C., Breen G., COVID Clinical Neuroscience Study Consortium (2022). Proteome-wide Mendelian randomization identifies causal links between blood proteins and severe COVID-19. PLoS Genet..

[9] Evans D.M., Davey Smith G. (2015). Mendelian Randomization: New Applications in the Coming Age of Hypothesis-Free Causality. Annu. Rev. Genomics Hum. Genet..

[10] Wainberg M., Sinnott-Armstrong N., Mancuso N., Barbeira A.N., Knowles D.A., Golan D., Ermel R., Ruusalepp A., Quertermous T., Hao K. (2019). Opportunities and challenges for transcriptome-wide association studies. Nat. Genet..

[11] Zhu H., Zhou X. (2021). Transcriptome-wide association studies: a view from Mendelian randomization. Quant. Biol..

[12] Yuan Z., Zhu H., Zeng P., Yang S., Sun S., Yang C., Liu J., Zhou X. (2020). Testing and controlling for horizontal pleiotropy with probabilistic Mendelian randomization in transcriptome-wide association studies. Nat. Commun..

[13] van der Harst P., Verweij N. (2018). Identification of 64 Novel Genetic Loci Provides an Expanded View on the Genetic Architecture of Coronary Artery Disease. Circ. Res..

[14] Zhang J., Dutta D., Köttgen A., Tin A., Schlosser P., Grams M.E., Harvey B., CKDGen Consortium, Yu B., Boerwinkle E. (2022). Plasma proteome analyses in individuals of European and African ancestry identify cis-pQTLs and models for proteome-wide association studies. Nat. Genet..

[15] Cohen J., Pertsemlidis A., Kotowski I.K., Graham R., Garcia C.K., Hobbs H.H. (2005). Low LDL cholesterol in individuals of African descent resulting from frequent nonsense mutations in PCSK9. Nat. Genet..

[16] Döring Y., van der Vorst E.P.C., Duchene J., Jansen Y., Gencer S., Bidzhekov K., Atzler D., Santovito D., Rader D.J., Saleheen D., Weber C. (2019). CXCL12 Derived From Endothelial Cells Promotes Atherosclerosis to Drive Coronary Artery Disease. Circulation.

[17] Brown A.A., Fernandez-Tajes J.J., Hong M.G., Brorsson C.A., Koivula R.W., Davtian D., Dupuis T., Sartori A., Michalettou T.-D., Forgie I.M. (2023). Genetic analysis of blood molecular phenotypes reveals common properties in the regulatory networks affecting complex traits. Nat. Commun..

[18] Sun B.B., Maranville J.C., Peters J.E., Stacey D., Staley J.R., Blackshaw J., Burgess S., Jiang T., Paige E., Surendran P. (2018). Genomic atlas of the human plasma proteome. Nature.

[19] Chick J.M., Munger S.C., Simecek P., Huttlin E.L., Choi K., Gatti D.M., Raghupathy N., Svenson K.L., Churchill G.A., Gygi S.P. (2016). Defining the consequences of genetic variation on a proteome-wide scale. Nature.

[20] Cupido A.J., Asselbergs F.W., Natarajan P., Ridker P.M., Hovingh G.K., Schmidt A.F., CHARGE Inflammation Working Group (2022). Dissecting the IL-6 pathway in cardiometabolic disease: A Mendelian randomization study on both IL6 and IL6R. Br. J. Clin. Pharmacol..

[21] Zheng J., Haberland V., Baird D., Walker V., Haycock P.C., Hurle M.R., Gutteridge A., Erola P., Liu Y., Luo S. (2020). Phenome-wide Mendelian randomization mapping the influence of the plasma proteome on complex diseases. Nat. Genet..

[22] Burgess S., Davey Smith G., Davies N.M., Dudbridge F., Gill D., Glymour M.M., Hartwig F.P., Holmes M.V., Minelli C., Relton C.L., Theodoratou E. (2020). Guidelines for performing Mendelian randomization investigations. Wellcome Open Res..

[23] Zuber V., Grinberg N.F., Gill D., Manipur I., Slob E.A.W., Patel A., Wallace C., Burgess S. (2022). Combining evidence from Mendelian randomization and colocalization: Review and comparison of approaches. Am. J. Hum. Genet..

[24] Giambartolomei C., Vukcevic D., Schadt E.E., Franke L., Hingorani A.D., Wallace C., Plagnol V. (2014). Bayesian test for colocalisation between pairs of genetic association studies using summary statistics. PLoS Genet..

[25] Hormozdiari F., van de Bunt M., Segrè A.V., Li X., Joo J.W.J., Bilow M., Sul J.H., Sankararaman S., Pasaniuc B., Eskin E. (2016). Colocalization of GWAS and eQTL Signals Detects Target Genes. Am. J. Hum. Genet..

[26] Hao K., Ermel R., Sukhavasi K., Cheng H., Ma L., Li L., Amadori L., Koplev S., Franzén O., d’Escamard V. (2022). Integrative Prioritization of Causal Genes for Coronary Artery Disease. Circ. Genom. Precis. Med..

[27] Li L., Chen Z., von Scheidt M., Li S., Steiner A., Güldener U., Koplev S., Ma A., Hao K., Pan C. (2022). Transcriptome-wide association study of coronary artery disease identifies novel susceptibility genes. Basic Res. Cardiol..

[28] Schmidt A.F., Finan C., Gordillo-Marañón M., Asselbergs F.W., Freitag D.F., Patel R.S., Tyl B., Chopade S., Faraway R., Zwierzyna M., Hingorani A.D. (2020). Genetic drug target validation using Mendelian randomisation. Nat. Commun..

[29] Chen L., Peters J.E., Prins B., Persyn E., Traylor M., Surendran P., Karthikeyan S., Yonova-Doing E., Di Angelantonio E., Roberts D.J. (2022). Systematic Mendelian randomization using the human plasma proteome to discover potential therapeutic targets for stroke. Nat. Commun..

[30] Sadler M.C., Auwerx C., Porcu E., Kutalik Z. (2021). Quantifying mediation between omics layers and complex traits. bioRxiv.

[31] Rosenson R.S., Hegele R.A., Fazio S., Cannon C.P. (2018). The Evolving Future of PCSK9 Inhibitors. J. Am. Coll. Cardiol..

[32] Ridker P.M., Rane M. (2021). Interleukin-6 Signaling and Anti-Interleukin-6 Therapeutics in Cardiovascular Disease. Circ. Res..

[33] Iuso A., Wiersma M., Schüller H.-J., Pode-Shakked B., Marek-Yagel D., Grigat M., Schwarzmayr T., Berutti R., Alhaddad B., Kanon B. (2018). Mutations in PPCS, Encoding Phosphopantothenoylcysteine Synthetase, Cause Autosomal-Recessive Dilated Cardiomyopathy. Am. J. Hum. Genet..

[34] Stein E.A., Mellis S., Yancopoulos G.D., Stahl N., Logan D., Smith W.B., Lisbon E., Gutierrez M., Webb C., Wu R. (2012). Effect of a Monoclonal Antibody to PCSK9 on LDL Cholesterol. N. Engl. J. Med..

[35] Interleukin-6 Receptor Mendelian Randomisation Analysis (IL6R MR) Consortium, Swerdlow D.I., Holmes M.V., Kuchenbaecker K.B., Engmann J.E., Shah T., Sofat R., Guo Y., Chung C., Peasey A. (2012). The interleukin-6 receptor as a target for prevention of coronary heart disease: a mendelian randomisation analysis. Lancet.

[36] Wang J., Pan W. (2020). The Biological Role of the Collagen Alpha-3 (VI) Chain and Its Cleaved C5 Domain Fragment Endotrophin in Cancer. OncoTargets Ther..

[37] Cescon M., Gattazzo F., Chen P., Bonaldo P. (2015). Collagen VI at a glance. J. Cell Sci..

[38] GTEx Consortium (2020). The GTEx Consortium atlas of genetic regulatory effects across human tissues. Science.

[39] Franzén O., Ermel R., Cohain A., Akers N.K., Di Narzo A., Talukdar H.A., Foroughi-Asl H., Giambartolomei C., Fullard J.F., Sukhavasi K. (2016). Cardiometabolic Risk Loci Share Downstream Cis- and Trans-Gene Regulation Across Tissues and Diseases. Science.

[40] SomaScan Menu https://menu.somalogic.com/.

[41] Wirka R.C., Wagh D., Paik D.T., Pjanic M., Nguyen T., Miller C.L., Kundu R., Nagao M., Coller J., Koyano T.K. (2019). Atheroprotective roles of smooth muscle cell phenotypic modulation and the TCF21 disease gene as revealed by single-cell analysis. Nat. Med..

[42] Turner A.W., Hu S.S., Mosquera J.V., Ma W.F., Hodonsky C.J., Wong D., Auguste G., Song Y., Sol-Church K., Farber E. (2022). Single-nucleus chromatin accessibility profiling highlights regulatory mechanisms of coronary artery disease risk. Nat. Genet..

[43] Oka C., Tsujimoto R., Kajikawa M., Koshiba-Takeuchi K., Ina J., Yano M., Tsuchiya A., Ueta Y., Soma A., Kanda H. (2004). HtrA1 serine protease inhibits signaling mediated by Tgfbeta family proteins. Development.

[44] Ron G., Globerson Y., Moran D., Kaplan T. (2017). Promoter-enhancer interactions identified from Hi-C data using probabilistic models and hierarchical topological domains. Nat. Commun..

[45] Rao S.S.P., Huntley M.H., Durand N.C., Stamenova E.K., Bochkov I.D., Robinson J.T., Sanborn A.L., Machol I., Omer A.D., Lander E.S., Aiden E.L. (2014). A 3D map of the human genome at kilobase resolution reveals principles of chromatin looping. Cell.

[46] Evrard S.M., Lecce L., Michelis K.C., Nomura-Kitabayashi A., Pandey G., Purushothaman K.-R., d’Escamard V., Li J.R., Hadri L., Fujitani K. (2016). Endothelial to mesenchymal transition is common in atherosclerotic lesions and is associated with plaque instability. Nat. Commun..

[47] Kovacic J.C., Dimmeler S., Harvey R.P., Finkel T., Aikawa E., Krenning G., Baker A.H. (2019). Endothelial to Mesenchymal Transition in Cardiovascular Disease: JACC State-of-the-Art Review. J. Am. Coll. Cardiol..

[48] Toma I., McCaffrey T.A. (2012). Transforming growth factor-β and atherosclerosis: interwoven atherogenic and atheroprotective aspects. Cell Tissue Res..

[49] Nielsen J.B., Rom O., Surakka I., Graham S.E., Zhou W., Roychowdhury T., Fritsche L.G., Gagliano Taliun S.A., Sidore C., Liu Y. (2020). Loss-of-function genomic variants highlight potential therapeutic targets for cardiovascular disease. Nat. Commun..

[50] Sadler M.C., Auwerx C., Deelen P., Kutalik Z. (2023). Multi-layered genetic approaches to identify approved drug targets. Cell Genom..

[51] Wishart D.S., Feunang Y.D., Guo A.C., Lo E.J., Marcu A., Grant J.R., Sajed T., Johnson D., Li C., Sayeeda Z. (2018). DrugBank 5.0: a major update to the DrugBank database for 2018. Nucleic Acids Res..

[52] Finan C., Gaulton A., Kruger F.A., Lumbers R.T., Shah T., Engmann J., Galver L., Kelley R., Karlsson A., Santos R. (2017). The druggable genome and support for target identification and validation in drug development. Sci. Transl. Med..

[53] Khetarpal S.A., Zeng X., Millar J.S., Vitali C., Somasundara A.V.H., Zanoni P., Landro J.A., Barucci N., Zavadoski W.J., Sun Z. (2017). A human APOC3 missense variant and monoclonal antibody accelerate apoC-III clearance and lower triglyceride-rich lipoprotein levels. Nat. Med..

[54] Zhang D.-W., Lagace T.A., Garuti R., Zhao Z., McDonald M., Horton J.D., Cohen J.C., Hobbs H.H. (2007). Binding of proprotein convertase subtilisin/kexin type 9 to epidermal growth factor-like repeat A of low density lipoprotein receptor decreases receptor recycling and increases degradation. J. Biol. Chem..

[55] Chong M., Sjaarda J., Pigeyre M., Mohammadi-Shemirani P., Lali R., Shoamanesh A., Gerstein H.C., Paré G. (2019). Novel Drug Targets for Ischemic Stroke Identified Through Mendelian Randomization Analysis of the Blood Proteome. Circulation.

[56] Bretherick A.D., Canela-Xandri O., Joshi P.K., Clark D.W., Rawlik K., Boutin T.S., Zeng Y., Amador C., Navarro P., Rudan I. (2020). Linking protein to phenotype with Mendelian Randomization detects 38 proteins with causal roles in human diseases and traits. PLoS Genet..

[57] Ghanbari F., Yazdanpanah N., Yazdanpanah M., Richards J.B., Manousaki D. (2022). Connecting Genomics and Proteomics to Identify Protein Biomarkers for Adult and Youth-Onset Type 2 Diabetes: A Two-Sample Mendelian Randomization Study. Diabetes.

[58] Hegele R.A. (2016). Multidimensional regulation of lipoprotein lipase: impact on biochemical and cardiovascular phenotypes. J. Lipid Res..

[59] Ference B.A., Robinson J.G., Brook R.D., Catapano A.L., Chapman M.J., Neff D.R., Voros S., Giugliano R.P., Davey Smith G., Fazio S., Sabatine M.S. (2016). Variation in PCSK9 and HMGCR and Risk of Cardiovascular Disease and Diabetes. N. Engl. J. Med..

[60] Bell A.S., Rosoff D.B., Mavromatis L.A., Jung J., Wagner J., Lohoff F.W. (2022). Comparing the Relationships of Genetically Proxied PCSK9 Inhibition With Mood Disorders, Cognition, and Dementia Between Men and Women: A Drug-Target Mendelian Randomization Study. J. Am. Heart Assoc..

[61] Rosoff D.B., Bell A.S., Jung J., Wagner J., Mavromatis L.A., Lohoff F.W. (2022). Mendelian Randomization Study of PCSK9 and HMG-CoA Reductase Inhibition and Cognitive Function. J. Am. Coll. Cardiol..

[62] Savić R., Yang J., Koplev S., An M.C., Patel P.L., O’Brien R.N., Dubose B.N., Dodatko T., Rogatsky E., Sukhavasi K. (2023). Integration of transcriptomes of senescent cell models with multi-tissue patient samples reveals reduced COL6A3 as an inducer of senescence. Cell Rep..

[63] Benjamins J.W., Yeung M.W., van de Vegte Y.J., Said M.A., van der Linden T., Ties D., Juarez-Orozco L.E., Verweij N., van der Harst P. (2022). Genomic insights in ascending aortic size and distensibility. EBioMedicine.

[64] McCulloch L.J., Rawling T.J., Sjöholm K., Franck N., Dankel S.N., Price E.J., Knight B., Liversedge N.H., Mellgren G., Nystrom F. (2015). COL6A3 Is Regulated by Leptin in Human Adipose Tissue and Reduced in Obesity. Endocrinology.

[65] Park J., Scherer P.E. (2012). Adipocyte-derived endotrophin promotes malignant tumor progression. J. Clin. Invest..

[66] Holm Nielsen S., Edsfeldt A., Tengryd C., Gustafsson H., Shore A.C., Natali A., Khan F., Genovese F., Bengtsson E., Karsdal M. (2021). The novel collagen matrikine, endotrophin, is associated with mortality and cardiovascular events in patients with atherosclerosis. J. Intern. Med..

[67] Chirinos J.A., Zhao L., Reese-Petersen A.L., Cohen J.B., Genovese F., Richards A.M., Doughty R.N., Díez J., González A., Querejeta R. (2022). Endotrophin, a Collagen VI Formation–Derived Peptide, in Heart Failure. NEJM Evid..

[68] Yoldemir S.A., Arman Y., Akarsu M., Altun O., Ozcan M., Tukek T. (2021). Correlation of glycemic regulation and endotrophin in patients with type 2 Diabetes; pilot study. Diabetol. Metab. Syndr..

[69] Scherer P.E., Gupta O.T. (2021). Endotrophin: Nominated for best supporting actor in the fibro-inflammatory saga. EBioMedicine.

[70] Sun K., Park J., Gupta O.T., Holland W.L., Auerbach P., Zhang N., Goncalves Marangoni R., Nicoloro S.M., Czech M.P., Varga J. (2014). Endotrophin triggers adipose tissue fibrosis and metabolic dysfunction. Nat. Commun..

[71] Jo W., Kim M., Oh J., Kim C.-S., Park C., Yoon S., Lee C., Kim S., Nam D., Park J. (2022). MicroRNA-29 Ameliorates Fibro-Inflammation and Insulin Resistance in HIF1α-Deficient Obese Adipose Tissue by Inhibiting Endotrophin Generation. Diabetes.

[72] Hara K., Shiga A., Fukutake T., Nozaki H., Miyashita A., Yokoseki A., Kawata H., Koyama A., Arima K., Takahashi T. (2009). Association of HTRA1 Mutations and Familial Ischemic Cerebral Small-Vessel Disease. N. Engl. J. Med..

[73] Uemura M., Nozaki H., Kato T., Koyama A., Sakai N., Ando S., Kanazawa M., Hishikawa N., Nishimoto Y., Polavarapu K. (2020). HTRA1-Related Cerebral Small Vessel Disease: A Review of the Literature. Front. Neurol..

[74] Winkler T.W., Grassmann F., Brandl C., Kiel C., Günther F., Strunz T., Weidner L., Zimmermann M.E., Korb C.A., Poplawski A. (2020). Genome-wide association meta-analysis for early age-related macular degeneration highlights novel loci and insights for advanced disease. BMC Med. Genomics.

[75] Ferrannini G., Manca M.L., Magnoni M., Andreotti F., Andreini D., Latini R., Maseri A., Maggioni A.P., Ostroff R.M., Williams S.A., Ferrannini E. (2020). Coronary Artery Disease and Type 2 Diabetes: A Proteomic Study. Diabetes Care.

[76] Fang H., ULTRA-DD Consortium, De Wolf H., Knezevic B., Burnham K.L., Osgood J., Lledó Lara A., Lledó Lara A., Kasela S., De Cesco S. (2019). A genetics-led approach defines the drug target landscape of 30 immune-related traits. Nat. Genet..

[77] Mostafavi H., Spence J.P., Naqvi S., Pritchard J.K. (2023). Systematic differences in discovery of genetic effects on gene expression and complex traits. Nat. Genet..

[78] Connally N.J., Nazeen S., Lee D., Shi H., Stamatoyannopoulos J., Chun S., Cotsapas C., Cassa C.A., Sunyaev S.R. (2022). The missing link between genetic association and regulatory function. Elife.

[79] Elenbaas J.S., Pudupakkam U., Ashworth K.J., Kang C.J., Patel V., Santana K., Jung I.-H., Lee P.C., Burks K.H., Amrute J.M. (2023). SVEP1 is an endogenous ligand for the orphan receptor PEAR1. Nat. Commun..

[80] Cuomo A.S.E., Nathan A., Raychaudhuri S., MacArthur D.G., Powell J.E. (2023). Single-cell genomics meets human genetics. Nat. Rev. Genet..

[81] Ferkingstad E., Sulem P., Atlason B.A., Sveinbjornsson G., Magnusson M.I., Styrmisdottir E.L., Gunnarsdottir K., Helgason A., Oddsson A., Halldorsson B.V. (2021). Large-scale integration of the plasma proteome with genetics and disease. Nat. Genet..

[82] Zhou D., Jiang Y., Zhong X., Cox N.J., Liu C., Gamazon E.R. (2020). A unified framework for joint-tissue transcriptome-wide association and Mendelian Randomization analysis. Nat. Genet..

[83] Zhao Z., Bi W., Zhou W., VandeHaar P., Fritsche L.G., Lee S. (2020). UK Biobank Whole-Exome Sequence Binary Phenome Analysis with Robust Region-Based Rare-Variant Test. Am. J. Hum. Genet..

[84] Hemani G., Zheng J., Elsworth B., Wade K.H., Haberland V., Baird D., Laurin C., Burgess S., Bowden J., Langdon R. (2018). The MR-Base platform supports systematic causal inference across the human phenome. Elife.

[85] Yavorska O.O., Burgess S. (2017). MendelianRandomization: an R package for performing Mendelian randomization analyses using summarized data. Int. J. Epidemiol..

[86] Wickham H. (2009).

[87] Pruim R.J., Welch R.P., Sanna S., Teslovich T.M., Chines P.S., Gliedt T.P., Boehnke M., Abecasis G.R., Willer C.J. (2010). LocusZoom: regional visualization of genome-wide association scan results. Bioinformatics.

[88] Ma W.F., Turner A.W., Gancayco C., Wong D., Song Y., Mosquera J.V., Auguste G., Hodonsky C.J., Prabhakar A., Ekiz H.A. (2022). PlaqView 2.0: A comprehensive web portal for cardiovascular single-cell genomics. Front. Cardiovasc. Med..

[89] Hao Y., Hao S., Andersen-Nissen E., Mauck W.M., Zheng S., Butler A., Lee M.J., Wilk A.J., Darby C., Zager M. (2021). Integrated analysis of multimodal single-cell data. Cell.

[90] Granja J.M., Corces M.R., Pierce S.E., Bagdatli S.T., Choudhry H., Chang H.Y., Greenleaf W.J. (2021). ArchR is a scalable software package for integrative single-cell chromatin accessibility analysis. Nat. Genet..

[91] Wang Y., Song F., Zhang B., Zhang L., Xu J., Kuang D., Li D., Choudhary M.N.K., Li Y., Hu M. (2018). The 3D Genome Browser: a web-based browser for visualizing 3D genome organization and long-range chromatin interactions. Genome Biol..

[92] Gusev A., Ko A., Shi H., Bhatia G., Chung W., Penninx B.W.J.H., Jansen R., de Geus E.J.C., Boomsma D.I., Wright F.A. (2016). Integrative approaches for large-scale transcriptome-wide association studies. Nat. Genet..

[93] Gamazon E.R., Wheeler H.E., Shah K.P., Mozaffari S.V., Aquino-Michaels K., Carroll R.J., Eyler A.E., Denny J.C., GTEx Consortium, Nicolae D.L. (2015). A gene-based association method for mapping traits using reference transcriptome data. Nat. Genet..

[94] Barbeira A.N., Dickinson S.P., Bonazzola R., Zheng J., Wheeler H.E., Torres J.M., Torstenson E.S., Shah K.P., Garcia T., Edwards T.L. (2018). Exploring the phenotypic consequences of tissue specific gene expression variation inferred from GWAS summary statistics. Nat. Commun..

[95] Burgess S., Dudbridge F., Thompson S.G. (2016). Combining information on multiple instrumental variables in Mendelian randomization: comparison of allele score and summarized data methods. Stat. Med..

