# Supplementary material for: Instrumental variable and colocalization analyses identify endotrophin and HTRA1 as potential therapeutic targets for coronary artery disease
Source: iScience. 2024 May 24;27(7):110104. doi: 10.1016/j.isci.2024.110104 (PMC11233907; doi:10.1016/j.isci.2024.110104)
Supplement: Supplementary file 1 — Document S1. Figures S1 and S2 [file mmc1.pdf]

iScience, Volume *27*

Supplemental information

Instrumental variable and colocalization analyses identify endotrophin and HTRA1 as potential therapeutic targets for coronary artery disease

Paul C. Lee, In-Hyuk Jung, Shreeya Thussu, Ved Patel, Ryan Wagoner, Kendall H. Burks, Junedh Amrute, Jared S. Elenbaas, Chul Joo Kang, Erica P. Young, Philipp E. Scherer, and Nathan O. Stitziel

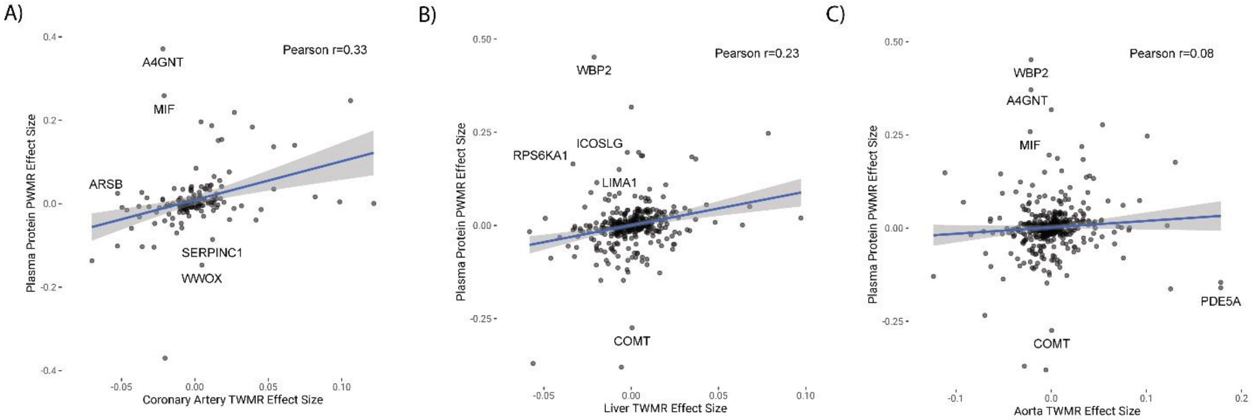

Figure S1. PWMR and TWMR Correlation, related to Figure 2. Correlated effect sizes of results from whole blood PWMR with TWMR from (**A**) coronary artery, (**B**) liver, or (**C**) aorta are plotted for each gene. Results with the largest directional discordance in effect sizes are labeled.

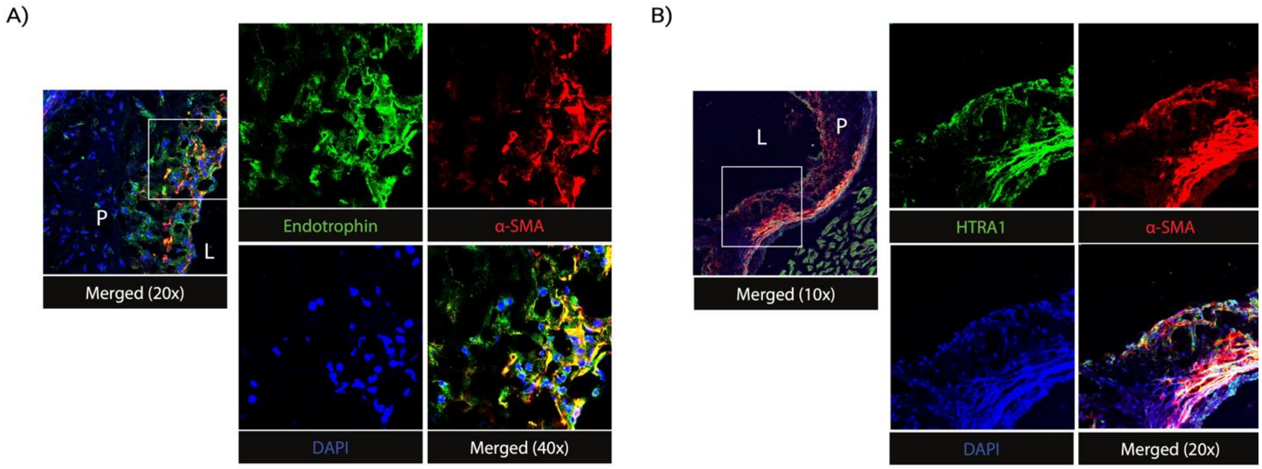

Figure S2. Endotrophin and HTRA1 expression in Mice Atherosclerotic Plaque, related to Figure 3. Immunohistochemistry of (**A**) endotrophin and (**B**) HTRA1 expression in murine aortic tissue. The outlined area in the left of each panel indicates the region magnified in the panels to the right. L=lumen; P=atherosclerotic plaque.
